# A shortcut to the thermodynamic limit for quantum many-body calculations of metals

**DOI:** 10.1038/s43588-021-00165-1

**Published:** 2021-12-16

**Authors:** Tina N. Mihm, Tobias Schäfer, Sai Kumar Ramadugu, Laura Weiler, Andreas Grüneis, James J. Shepherd

**Affiliations:** 1 Department of Chemistry, University of Iowa, Iowa City, Iowa USA; 2 Institute for Theoretical Physics, TU Wien, Vienna, Austria

**Keywords:** Method development, Quantum chemistry, Computational methods, Density functional theory

## Abstract

Computationally efficient and accurate quantum mechanical approximations to solve the many-electron Schrödinger equation are crucial for computational materials science. Methods such as coupled cluster theory show potential for widespread adoption if computational cost bottlenecks can be removed. For example, extremely dense *k*-point grids are required to model long-range electronic correlation effects, particularly for metals. Although these grids can be made more effective by averaging calculations over an offset (or twist angle), the resultant cost in time for coupled cluster theory is prohibitive. We show here that a single special twist angle can be found using the transition structure factor, which provides the same benefit as twist averaging with one or two orders of magnitude reduction in computational time. We demonstrate that this not only works for metal systems but also is applicable to a broader range of materials, including insulators and semiconductors.

## Abstract (web summary)

The authors demonstrate an effective approach to lower the computing time required to accurately reach the thermodynamic limit in quantum many-body calculations. This method can be applied to solve problems in a wide range of material systems, including metals, insulators and semiconductors.

## Main

Electron correlation in metals has been a research interest for much of the past century. Accurate total energy evaluation in metals has therefore been an important milestone to demonstrate the generalizability of electronic structure methods. When it comes to many-body perturbation theories, methods that show potential include those that perform resummations to improve accuracy and numerical stability^1–5^. One such method is coupled cluster theory, and its success in molecular quantum chemistry has led many to start developing these same tools for solids^6–15^. A crucial barrier to the widespread adoption of these methods for calculations in solids is the finite-size errors that can arise when simulating the bulk limit of a material with a supercell^7^. A method that can overcome this barrier would come to play a central role in understanding and predicting chemical processes in the gas phase, condensed matter systems and on surfaces.

One hurdle on the journey to a universal application of periodic coupled cluster theory to all solids is applying the above successes to real metals. The quintessential property of a metal—that it has a zero gap—has led to the view of coupled cluster calculations being intractable. This view comes from experiences in molecular systems with small gaps between the highest occupied molecular orbital (HOMO) and lowest unoccupied molecular orbital (LUMO); such systems are prone to strong correlation effects that are inaccessible by single reference methods such as coupled cluster theories. However, simulations of metals are also notorious for requiring larger supercells than those of insulators. This allows for more dense *k*-point grids to model the effects of long-range correlation. An alternative to denser grids is to use twist averaging^16,17^, which averages the energy over all *k*-point grid offsets (called twist angles); however, larger supercells and twist averaging both have a prohibitive cost in computational time.

Here we show that the twist-averaged energy can be found without the need to average over grid offsets directly, with a one or two orders of magnitude reduction in computational time. We do so by finding a special twist angle using the transition structure factor^9^, which is a map of electronic excitations from the Hartree–Fock (HF) wavefunction. We also show that this was a key bottleneck in converging coupled cluster theory calculations to the thermodynamic limit (TDL) by showing examples of coupled cluster theory singles and doubles (CCSD) calculations applied to insulators, semiconductors and metals. We also converge the TDL and complete basis set limit total energies for two metallic phases of lithium and the semiconductor-to-metal transition in silicon. For silicon, we compare our results with other calculations and experimental measurements of the transition pressure^18–24^. Overall, this paper demonstrates that the success found in the recent treatments of the uniform electron gas^25–35^ can be transferred to solids.

## Results

### Structure-factor-based twist averaging method

**Fig. 1:**
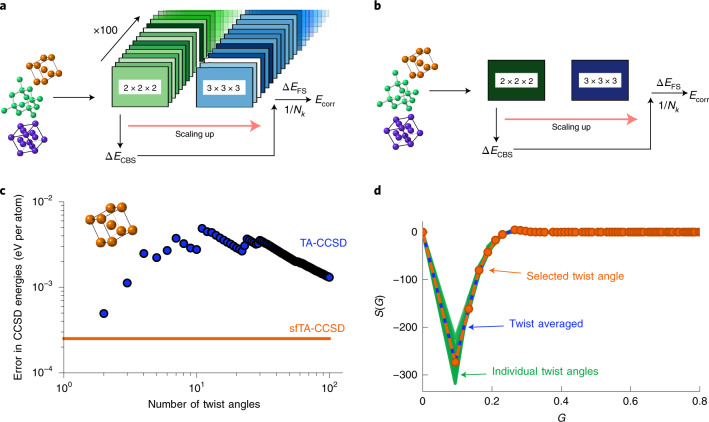
Illustrations of sfTA with computational efficiency data. **a**, A solid-state calculation flow scheme emphasizing where 100 twist angles are used. **b**, A solid-state calculation flow scheme with one twist angle chosen by sfTA. **c**, A simple example of convergence of a regular twist-averaging stochastic error for sodium. The sfTA difference line represents systematic error from the twist average. **d**, Different treatments for the CCSD transition structure factor, *S*(*G*), for sodium plotted against momentum, *G*. Green, 100 twist angles; blue, the average over the same twist angles; orange, the sfTA twist angle obtained from 100 MP2 calculations. Source data

As described above, a computational time cost bottleneck in converging coupled cluster theory calculations to the bulk limit originates from the required density of points used to sample the first Brillouin zone by means of a twist-averaging procedure. Each point is a momentum vector used to offset *k*-point grids. The key idea of our approach is based on the premise that the electronic transition structure factor can be used to find a single special offset for coupled cluster theory calculations, achieving the same accuracy as with the twist-averaging procedure. This is based on using a method that uses less computer time than CCSD to calculate a twist-averaged structure factor and choosing a specific twist from the same set of random offsets to best match the average (this is summarized below with additional details in the Methods). In doing so, the number of required coupled cluster theory calculations can be reduced by one or two orders of magnitude depending on how many twist angles were required for the original twist average. Figure 1a,b illustrate this idea schematically in the workflow of a typical solid-state calculation.

The results of applying our procedure is shown in an illustrative example (Fig. 1c,d) of the transition structure factor of sodium metal. Throughout this paper we used second-order Møller–Plesset perturbation theory (MP2) and CCSD theory as the low- and high-level methods, respectively. When twist averaging CCSD (Fig. 1c), it can be seen that the error is not converged (indicating undersampling) for fewer than 40 twist angles. When our sfTA scheme is used, the systematic error is of the order 1 meV el^–1^. By contrast, the stochastic error in twist averaging only reached this value when over 100 twist angles were used (noting here that the reference value used to obtain this error is the twist-averaged CCSD (TA-CCSD) energy at 100 twist angles). In Fig. 1d, it can be seen that the selected twist angle found through MP2 calculations is successful in reproducing the TA-CCSD structure factor from one CCSD calculation (mean absolute error = 0.08(13)). This small error demonstrates that one twist angle is able to reproduce the twist-averaged transition structure factor. We refer to this method as structure-factor-based twist averaging (sfTA) due to the way it reproduces the twist-averaged energy on the basis of the best representation of the transition structure factor.

**Fig. 2:**
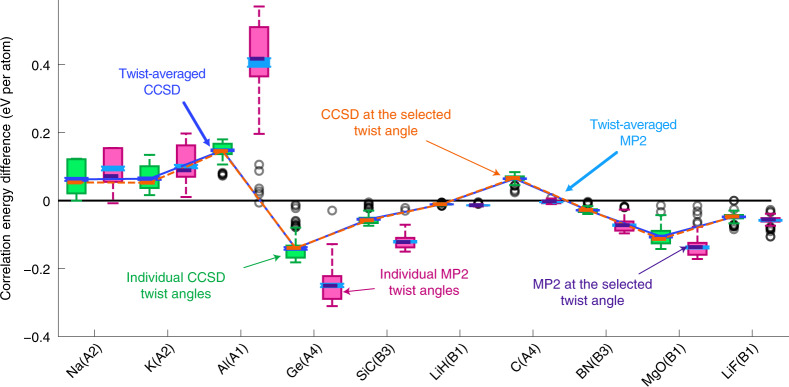
Energy differences to Γ-centered grid for twist averaging and sfTA for a range of systems. The energies for the 100 random twist angles have been graphed as box-and-whisker plots. Twist-averaged CCSD and sfTA-CCSD results are overlaid and joined by a line to help guide the eye. The MP2 data come from an intermediate stage in the sfTA calculation before the sfTA-selected twist angle is transferred to a CCSD calculation. The *x*-axis labels systems ordered with increasing experimental bandgap (left to right)^58^ and Strukturbericht symbols are used (details in Methods). Boxes in the box-and-whisker plots represent the lower (Q1) and upper (Q3) quartiles of energies; the top and bottom whiskers are *Q*_1_ − 1.5(*Q*_3_ − *Q*_1_) and *Q*_3_ + 1.5(*Q*_3_ − *Q*_1_), respectively, where *Q*_*i*_ is the *i*th quartile. Gray circles are outliers, which are more than 1.5(*Q*_3_ − *Q*_1_) away from the nearest *Q*_*i*_. All twist-averaged energies are plotted with error bars representing one standard error; these are sometimes small and hidden by the marker. Source data

The protocol to run sfTA-CCSD is therefore: (1) to run HF and MP2 at 100 randomly generated twist angles (random twist angles were chosen due to the findings of previous studies^17^); (2) average the transition structure factor and select a twist angle whose transition structure factor best matches the average (according to equation (7)); (3) run a CCSD calculation at the selected twist angle. We test this for ten systems to validate our protocol (including metals, semiconductors and insulators) by comparing the result of the sfTA-CCSD with TA-CCSD (from 100 randomly generated twist angles). The results of this are shown in Fig. 2, showing good agreement between sfTA-CCSD and TA-CCSD with a mean difference of 0.003(2) eV per atom averaged across the ten systems (number in parentheses is one standard error).

### Total energy calculations

A more practical demonstration of how this method works comes from studying the energy differences between material phases, which requires scrupulous accounting for finite basis set, as well as finite system size, errors. We here present a summary of our protocol (with concrete examples available in the ‘Assembling the total energy’ section in the Supplementary Information, as well as Supplementary Tables 1 and 2) in which: (1) we run sfTA-CCSD calculations at increasingly dense *k*-point meshes, keeping the number of orbitals (*M*) per *k*-point constant. (2) Calculations using natural orbitals are performed for a small *k*-point mesh (for example, 2 × 2 × 2), which are then converged to the complete basis set (CBS) limit using an extrapolation with a 1/*M* power law. The following correction is then found and applied: Δ*E*_CBS_(222) = *E*_CBS_(222) − *E*_corr_(*M*, 222), where 222 indicates that a 2 × 2 × 2 mesh is employed throughout; *E*_CBS_(222) and *E*_corr_(*M*, 222) are the correlation energies at the complete basis set (CBS) limit and with *M* basis functions per *k*-point, respectively. (3) We apply finite-size corrections, Δ*E*_FS_, to each mesh using an interpolation scheme^7^. (4) We extrapolate the resultant correlation energies to the TDL using a power-law of 1/*N*_*k*_ (where *N*_*k*_ is the number of *k*-points) to remove residual finite-size errors. (5) Finally, to find the total energy, the correlation energy from steps 1–4 is added to HF calculations, which have been extrapolated to the TDL. As HF is considerably less expensive than CCSD, larger *k*-point meshes (up to 6 × 6 × 6) are used for this step. Two parameters have to be chosen in this protocol: the small *k*-point grid (referenced above as 2 × 2 × 2) and *M*. These are chosen to be the largest reasonable choice within computer time restrictions. Here we wanted these calculations to finish within approximately one day (on an eight-core Intel(R) Xeon(R) CPU E5-2680 v4 2.40 GHz processor). In practice, the resulting correction is relatively insensitive to this choice^29^. Details on the parameters used for this study are given in the Methods.

### Energy difference between two phases of lithium

**Fig. 3:**
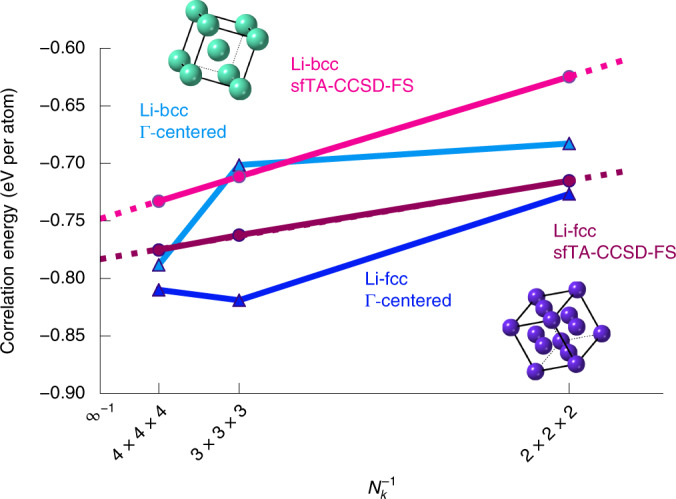
sfTA-CCSD-FS applied to two phases of lithium. A comparison between the sfTA-CCSD with finite-size correction (sfTA-CCSD-FS) and Γ-centered grid CCSD energies is shown for fcc and bcc phases of lithium. A 1/*N*_*k*_ axis is used to emphasize convergence to the TDL (going from right to left); *N*_*k*_ is the number of *k*-points (that is, eight for 2 × 2 × 2). Linear extrapolations to the TDL are shown as dashed lines. Source data

Figure 3 shows the difference between the correlation energies for the face- (fcc) and body-centered cubic (bcc) phases of lithium. This figure shows the extrapolation to the TDL of sfTA-CCSD correlation energies from a variety of *k*-point meshes. Each point has been corrected to the complete basis set limit. Overall, the smoothness of this convergence to the TDL seen in Fig. 3 when compared with the calculation from a Γ-centered grid (with a twist angle offset of zero) shows a successful application of sfTA.

The TDL HF was then obtained by extrapolating the twist-averaged Hartree–Fock (TA-HF) energies to the TDL (using *k*-point meshes up to 6 × 6 × 6). The HF energy difference in the TDL is 0.015(5) eV per atom with bcc having the lower energy, where the error in parentheses comes from the standard error in the fit parameters from extrapolation. Adding this to the correlation energy result from Fig. 3, which is −0.0350(9) eV per atom (with fcc lower), yields −0.020(5) eV per atom. This number indicates that the electronic contribution at 0 K slightly favors fcc and the number is also statistically significant with respect to extrapolation error. It is also noteworthy that the HF and correlation energy contributions are opposite in sign.

### Equation-of-state curves for silicon

We now apply sfTA to the energy–volume curves of silicon in a diamond lattice and β-tin (Sn) lattice. These allotropes of silicon are semiconducting and metallic, respectively, constituting an excellent test case for the ability of the proposed sfTA technique to treat small-gap systems on the same footing. We performed calculations using sfTA-CCSD on a 2 × 2 × 2 and 3 × 3 × 3 *k*-point mesh. A complete basis-set correction was derived from a 2 × 2 × 2 *k*-point mesh run at each volume^7^. Finite-size corrections were applied using a mesh-interpolation scheme and the resultant energies extrapolated using 1/*N*_*k*_ as for lithium above. The correlation energies were then added to TDL-extrapolated TA-HF energies (using *k*-point meshes up to 5 × 5 × 5) to obtain total energies.

**Fig. 4:**
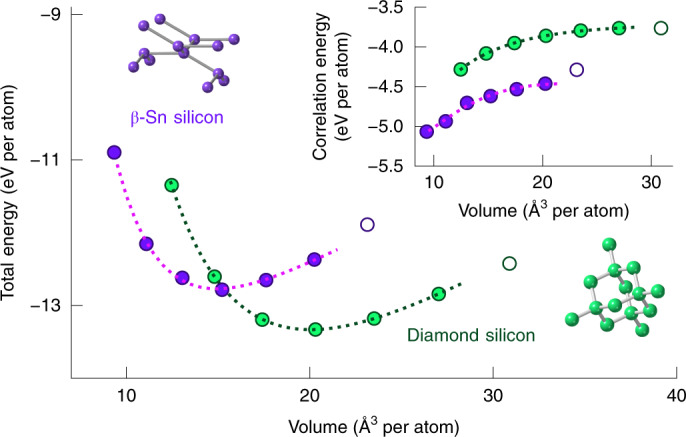
Energy–volume curve using sfTA-CCSD for the diamond and β-Sn phases of silicon. The main panel shows total energies and the inset shows correlation energies. The dashed lines show a fit to the Birch–Murnaghan equation of state for the diamond and β-Sn phases. The fit was used to obtain the lattice properties of the phases shown in Table 1. To make a fair comparison with previous results for the fit, points represented by open circles were omitted (see ‘Results’ section). A core-polarization correction has been added (Methods). Source data

The Birch–Murnaghan equation of state was fit to the diamond and β-Sn phases of silicon, shown in Fig. 4, allowing us to obtain lattice properties including equilibrium volumes and bulk moduli. We can clearly see the minima for both phases, with diamond reaching a minimum at around 20 Å^3^ per atom and *β*-tin reaching a minimum at approximately 15 Å^3^ per atom. The transition between the two phases can clearly be seen to take place between volumes of around 15–18 Å^3^ per atom. In Table 1 the fitting parameters are compared with several high-accuracy quantum Monte Carlo methods from previous studies^18–21^ and experimental values^22–24^.

**Table 1:** Lattice properties obtained from the Birch–Murnaghan equation of state for the diamond and β-Sn phases of silicon

Structure	Property	TA-HF	sfTA-CCSD-FS	sfTA-DCSD-FS	Experiments	DMC^a^	DMC^b^	DMC + EMP-pp^c^	AFQMC
D-Si	*V*_t_ (Å^3^ per atom)	15.52	17.62	–	18.15	–	18.14	17.83	18.15
	*B*_0_ (GPa)	104	105.1	–	99.2	103.0	98.0	96.2	–
	${B}_{0}^{\prime}$	3.82	3.83	–	4.11	–	4.6	4.19	–
	*V*_0_ (Å^3^ per atom)	20.78	20.04	–	20.0	20.11	19.98	19.75	–
β-Sn Si	*V*_t_ (Å^3^ per atom)	12.16	13.5	–	13.96	–	13.9	13.81	13.955
	*B*_0_ (GPa)	112	118.3	–	–	114	107	104.2	–
	${B}_{0}^{\prime}$	4.05	4.6	–	–	–	4.6	4.7	–
	*V*_0_ (Å^3^ per atom)	15.96	14.95	–	–	15.26	15.2	15.17	–
	Δ*E* (eV per atom)	1.302	0.562	0.494	–	0.505	0.424	0.329	0.365^d^
	*P*_t_ (GPa)	52.96	17.37	15.26	–	17.8	15.3	13.16	13.9
	*P*_t_ vib. (GPa)	51.66	16.07	13.96	11.3–12.5	16.5	14.0	12.2	12.6

Lattice properties for the two silicon phases, including the transition volume (*V*_t_), bulk modulus (*B*_0_), pressure derivative of the bulk modulus ($B_0$), volume at equilibrium (*V*_0_), energy difference between the two minima (Δ*E*), transition pressure (*P*_t_) and fully corrected transition pressures (*P*_t_ vib.) (see Methods for details). Our TA-HF and sfTA-CCSD energies are compared with those from DMC, AFQMC and experiments. The following superscripts distinguish between different DMC studies: ^a^Alfè et al.^19^, ^b^Hennig et al.^18^ and ^c^Maezono et al.^21^. AFQMC numbers come from Purwanto et al.^20^ and to obtain ^d^ we applied a core-polarization correction ourselves. The column marked DMC + EMP-pp used an empirical pseudopotential correction (EMP-pp). The energies and transition pressures consistently include core-polarization contributions (see Methods for details). Experimental numbers come from various sources^22–24^ as organized by Hennig and colleagues^18^.

We note that the diffusion quantum Monte Carlo (DMC) findings compiled in Table 1 span a relatively large range for different equilibrium properties including equilibrium volumes, as well as bulk moduli and their derivatives. The equilibrium volumes of silicon diamond in particular range from 19.75 Å^3^ per atom to 20.11 Å^3^ per atom, whereas the experimental equilibrium volume was measured to be 20.00 Å^3^ per atom. The difference in the DMC estimates can partly be explained by different approximations used to correct finite-size errors and the dependence on the employed pseudopotentials. Similar trends as seen for the equilibrium volumes can also be observed for the bulk moduli. The most striking changes between the different DMC calculations can be observed for the energy differences between both phases, which reduces from 505 meV per atom^19^ to 329 meV per atom^21^. Due to the strong dependence of the transition pressure between the diamond to β-Sn allotropes on the energy difference, the predictions change from 17.8 GPa to 13.16 GPa, which is a gradual improvement compared with the experimental estimates that lie between 11.2 and 12.6 GPa. This is corroborated by an independent study using auxiliary-field quantum Monte Carlo (AFQMC), which found numbers in good agreement with the spread of DMC results^20^.

We now turn to the discussion of results obtained from coupled cluster theory. Recalling that HF (rather than density functional theory (DFT)) is used as a starting point for coupled cluster theory calculations (HF fit parameters are listed in Table 1). These show that HF theory strongly overestimates the diamond equilibrium volume and bulk modulus compared with experiment and DMC, which can mainly be attributed to the neglect of electronic correlation effects. Likewise, the energy difference between the diamond and β-Sn phase is greatly overestimated, yielding a transition pressure that is almost five times larger than the experimental findings. Even with this starting point, CCSD theory is generally able to improve on the HF description by accounting for correlation effects (once we have accounted for finite-size as well as basis-set corrections).

We calculated a CCSD energy difference of 0.562 eV per atom between the two phases, which was found to be larger than the largest DMC energy difference (0.505 eV per atom) by 0.057 eV per atom. The transition pressure, by contrast, is 17.37 GPa and lies within the region spanned by DMC. The remaining discrepancy between CCSD and DMC is not surprising. Although CCSD forms an important step towards chemical accuracy, it is known from quantum chemical wavefunction calculations of molecules that the inclusion of perturbative triple particle-hole excitation operators (for molecular and insulating systems) is needed for chemically accurate reaction energies^36^. However, we have previously shown that coupled cluster single and doubles with perturbative triples, CCSD(T), is divergent due to its perturbative component^33^. One alternative possibility to improve on CCSD theory has recently arisen in the literature with the distinguishable cluster theory (DCSD)^37^. When we run this at the mid-point volume we find that DCSD lowers the energy gap between the phases by 68 meV per atom to 494 meV per atom. If this were uniform across the volume curve, this is equivalent to lowering the transition pressure to 15.26 GPa. This is promising as CCSD is the simplest correction to HF in the coupled cluster hierarchy of methods and these calculations took only four days (per volume point, including every component of the calculation) on a maximum number of 16 cores (on an Intel(R) Xeon(R) Platinum 8168 Processor 3.1/3.9 turbo GHz processor).

Coupled cluster theory singles and doubles theory has a more mixed performance with the other parameters. The transition volumes are in reasonable agreement with DMC, but the bulk moduli are not improved relative to HF theory. The pressure derivative of the bulk modulus improved in β-Sn but not in diamond. We found that the parameters were sensitive to the region of the curve that we fit and it is possible that our uniform sampling of the curve is the cause of the discrepancies (as the DMC studies used more points around the equilibrium volume).

Taken together, these numbers are in reasonable agreement with the DMC results. Overall, we note a balanced treatment of correlation between the phases using the same protocol (that is we did not have to change anything in our protocol to move between a semiconductor and a metal). The improvement from CCSD to DCSD also demonstrates the way in which quantum chemical wavefunction methods can be improved. In closing, we do agree with the DMC study by Alfè et al.^19^, who conclude that the numbers from electronic structure calculations were only in agreement with experiment after accounting for vibrational and anharmonic effects. In practice, this means that the structural transition pressure should be a little higher than experiment if the electronic structure is being treated appropriately.

## Discussion

Periodic coupled cluster theory benefits from an approach to select a twist angle more than quantum Monte Carlo for two important reasons. The first is that basis-set incompleteness error from a twist-averaged (or balanced) description of correlation commutes more reliably with the electron number. This provides the crucial benefit of reducing the basis-set size required to treat metals and materials with small bandgaps. The second is that TDL corrections depend on the same relationship, and the increased commutivity also helps these corrections become more consistent. Taken together, the small offset in the *k*-point grid means calculations that previously were prohibitively costly in terms of computer time are now routinely feasible.

This study generalizes our past work which selected twist angles in the uniform electron gas^28,30^, making it work for real materials including metals. In those works, we considered the Baldereschi point^38^ as a candidate for the single twist offset and found it to be unsuccessful for our purposes. There have also been more recent special twist angles proposed by the QMC community^39,40^. In particular, where the approach by Degrada and colleagues^40^ focuses on minimizing the one-body overall finite-size error in a mean-field wavefunction by way of its energy, we are focused on minimizing the two-body finite-size error in a correlated wavefunction property. This is because we wish to stay within the canonical coupled cluster formalism to allow for finite-size corrections and extrapolation to the TDL (Fig. 1). Overall, we believe this paper describes a generalized approach for twist angle selection for coupled cluster theory.

We note that a range of DFT calculations of the transition pressure of silicon employing approximate exchange-correlation functionals have been performed by Xiao and co-workers^41^. Their findings show that DFT transition pressures often agree excellently with experiment and perform very well in terms of their trade-off between accuracy and computational cost, though performance varies considerably across different functionals. Our long-term goal would be to benchmark these functionals without having to rely on experiment and to make explicit comparisons with local cluster methods^12,42^.

An important limitation of the study is that we found the parameters derived for the silicon phase transition to be relatively sensitive to the energy and volume points of the fit (Fig. 4 and Table 1); thus, as there are different twist angles chosen for each point across the energy–volume curve, it is possible that this contributes to the remaining discrepancies between our calculations and those from other methods in a way that is indistinguishable from the intrinsic accuracy of CCSD. We were not able to investigate this here through direct comparison between twist averaging and sfTA (across the volume curve) due to the cost in computational time of twist averaging, and this will be the topic of a future study. Another limitation of our study is that we know that a triples (or beyond) treatment would be required for benchmarks to identify optimal DFT functionals^43^. Which approximation to the perturbative triple particle-hole excitation operators achieves CCSD(T)-quality correlation energy for metals remains an open question at this point^33^. We note, however, that renormalized coupled cluster approaches^44^ could be well-suited for such situations and will be explored in future work. We believe that the selected twist angle method presented here will transfer well to higher levels of theory, due to results on twist angle selection for model systems^30^.

## Methods

### The Hamiltonian

The quantum mechanical many-electron Hamiltonian of periodic solids is invariant under any translational symmetry transformation that respects the periodicity of the attractive nuclear potential. Crystal momentum vectors **k** that lie in the first Brillouin zone serve as a label for translational symmetry transformations. The full Hamiltonian can be written as

1 $$\hat{H}=\int {{{\rm{d}}}}{{{\bf{k}}}}\left\{{\hat{h}}_{1}({{{\bf{k}}}})+\iiint {{{\rm{d}}}}{{{\bf{k}}}}^{\prime} {{{\rm{d}}}}{{{\bf{k}}}}^{\prime\prime} {{{\rm{d}}}}{{{\bf{k}}}}^{\prime\prime\prime} {\hat{h}}_{2}\left({{{\bf{k}}}},{{{\bf{k}}}}^{\prime} ,{{{\bf{k}}}}^{\prime\prime} ,{{{\bf{k}}}}^{\prime\prime\prime} \right)\right\},$$

where the one- and two-electron Hamiltonians for a particular set of *k*-vectors are given in second quantization by

2 $${\hat{h}}_{1}({{{\bf{k}}}})=\mathop{\sum}\limits_{p({{{\bf{k}}}}),q({{{\bf{k}}}})}{h}_{pq}{a}_{p}^{{\dagger} }{a}_{q}$$

and

3 $${\hat{h}}_{2}({{{\bf{k}}}},{{{\bf{k}}}}^{\prime} ,{{{\bf{k}}}}^{\prime\prime} ,{{{\bf{k}}}}^{\prime\prime\prime} )=\frac{1}{2}\mathop{\sum}\limits_{p({{{\bf{k}}}}),q({{{\bf{k}}}}^{\prime} )}\mathop{\sum}\limits_{r({{{\bf{k}}}}^{\prime\prime} ),s({{{\bf{k}}}}^{\prime\prime\prime} )}\langle pq| rs\rangle {a}_{p}^{{\dagger} }{a}_{q}^{{\dagger} }{a}_{r}{a}_{s},$$

respectively. The indices *p*, *q*, *r* and *s* refer to occupied and unoccupied one-electron Bloch states characterized by **k**; ${a}_{p}^{{\dagger} }$ and $a_q$ are creation and annihilation operators, respectively. The two-electron integrals 〈*pq*∣*rs*〉 are non-zero only if the corresponding *k*-vectors conserve total crystal momentum. In practice, the TDL—computed by integrating over all **k**—is approached by sampling the first Brillouin zone using a regular *k*-mesh with an increasing number of *k*-points, *N*_*k*_. In this study, we focus on the way in which finite-size effects (from these *k*-meshes) arise and can be remedied within a canonical periodic coupled cluster formalism with delocalized Bloch orbitals. We recognize that there are alternative methods, which have been designed to circumvent finite-size effects in other coupled cluster approaches including local cluster approximations^12,42^, incremental schemes^13–15^ and embedding^45–49^. We believe a canonical approach can be complementary to these methods, especially if finite-size effects can be removed for metals as we demonstrate here.

Here, by choosing a special shift to the employed *k*-mesh, we are effectively choosing one Hamiltonian of a specific symmetry that can also be applied to metals and—in combination with recently proposed finite-size corrections^7,50^—yields an even more rapid and numerically stable convergence of correlation energies to the TDL. To this end, we approximate the Brillouin zone integrations in equation (1) such that $\int{\mathrm{d}}{{{\bf{k}}}}F({{{\bf{k}}}})\approx \mathop{\sum }\nolimits_{i = 1}^{{N}_{k}}\frac{1}{{N}_{k}{{\Omega }}}F({{{{\bf{k}}}}}_{i}+{{{{\bf{k}}}}}_{s})$. Here, *F*(**k**) refers to the terms inside the curly brackets in equation (1). The vector **k**_s_ corresponds to an offset of the employed *k*-mesh with respect to the origin and is referred to as a twist angle or offset throughout this work. This shifting of the *k*-mesh can cause the orbital occupations to change and, when they do, this changes the character of the reference to yield a substantial change in the coupled cluster wavefunction. By contrast, in conventional twist-averaging, a large number of **k**_s_ vectors are drawn at random or from a grid and quantities are averaged over these points. The vectors are the set of unique offsets which can be made to the *k*-mesh which lie in the simulation-cell Brillouin zone.

### Structure factor twist averaging

Our approach is to select one twist angle at which to perform the calculation by considering the twist angle that best represents the twist-averaged electronic transition structure factor computed from the amplitudes of the first-order perturbed wavefunction used in MP2 theory. This is represented schematically in Fig. 1.

The Fourier coefficients of the transition structure factor can be used to express the projected correlation energy (*E*_corr_) obtained from a perturbed wavefunction $\left|{{\varPsi }}\right\rangle$ such that

4 $${E}_{{{{\rm{corr}}}}}=\langle {{{\varPsi }}}_{{{{\rm{HF}}}}}| H-{E}_{{{{\rm{HF}}}}}| {{\varPsi }}\rangle =\mathop{\sum}\limits^{\prime} v({{{\bf{G}}}})S({{{\bf{G}}}}).$$

where *Ψ*_HF_ and *E*_HF_ are the HF wavefunction and HF energy, respectively. Here the momentum **G** corresponds to a plane wave vector that is defined as **G** = **g** + Δ**k**, where **g** is a reciprocal lattice vector and Δ**k** is the difference between any two crystal momentum vectors that are conventionally chosen to sample the first Brillouin zone; *v*(**G**) are the Fourier coefficients of Coulomb kernel with the familiar form of $\frac{4\pi }{{G}^{2}}$ for excitations allowed by momentum conservation, and the prime on the sum implies that the **G** = 0 contribution is treated in an approximate fashion^7,9^.

To express the transition structure factor in terms of quantities computed by coupled cluster theory, we follow the notation of Liao and Grüneis^9^ for mathematical consistency. The coupled cluster wavefunction has amplitudes that can be written ${T}_{ij}^{ab}={t}_{ij}^{ab}+{t}_{i}^{a}{t}_{j}^{b}$. Here, the *i* and *j* indices refer to occupied orbitals, whereas *a* and *b* indices refer to unoccupied orbitals; ${t}_{i}^{a}$ and ${t}_{ij}^{ab}$ are singles and doubles amplitudes, respectively. The transition structure factor can be recast as:

5 $$\begin{array}{ll}S({{{\bf{G}}}})=\mathop{\sum}\limits_{{{{{\bf{k}}}}}_{i},{{{{\bf{k}}}}}_{j},{{{{\bf{k}}}}}_{a}}\mathop{\sum}\limits_{{n}_{i},{n}_{j},{n}_{a},{n}_{b}}{T}_{ij}^{ab}\left[2{C}_{i}^{a}({{{\bf{G}}}}){C}_{b}^{j* }({{{\bf{G}}}})\right.\left.-{C}_{i}^{b}({{{\bf{G}}}}){C}_{a}^{j* }({{{\bf{G}}}})\right],\end{array}$$

The coefficients ${C}_{i}^{a}({{{\bf{G}}}})$ arise from the Fourier transform of the co-densities of two orbitals *i* and *a* and the electron repulsion integrals can also be written in terms of these intermediates^9^. We note that this uses a mixed estimator formalism as in equation (4). When the structure factor is calculated from an MP2 calculation, the amplitudes come from the first-order perturbed wavefunction defined by ${t}_{ij}^{ab}={v}_{ij}^{ab}/({\epsilon }_{i}+{\epsilon }_{j}-{\epsilon }_{a}-{\epsilon }_{b})$ (where *ϵ* are HF eigenvalues and ${v}_{ij}^{ab}$ are electron repulsion integrals). The ${t}_{i}^{a}{t}_{j}^{b}$ terms are zero for MP2 due to Brillouin’s theorem.

From this, we can define a twist-averaged transition structure factor as follows:

6 $$\overline{S}({{{\bf{G}}}})=\frac{1}{{N}_{\mathrm{s}}}\mathop{\sum }\limits_{{{{{\bf{k}}}}}_{\mathrm{s}}}^{{N}_{\mathrm{s}}}{S}_{{{{{\bf{k}}}}}_{\mathrm{s}}}({{{\bf{G}}}})$$

where *N*_s_ is the number of twist angles. This structure factor would then be expected to exhibit fewer finite-size effects in common with the twist-averaged energy. The benefit of such a transition structure factor is twofold. First, using $\overline{S}({{{\bf{G}}}})$ in equation (4) would lead to an improved energy estimate. Second, a finite-size correction^9^, which we will be using based on $\overline{S}({{{\bf{G}}}})$, would be similarly improved. To circumvent the cost in computational time of evaluating ${\sum }_{{{{{\bf{k}}}}}_{\mathrm{s}}}$ directly for CCSD, we instead find one twist angle that most effectively approximates the twist-averaged transition structure factor using MP2 theory. To do this, we find the twist angle, which minimizes the residual

7 $$r=\mathop{\sum}\limits_{{{{\bf{G}}}}}{\left|\overline{S}({{{\bf{G}}}})-{S}_{{{{{\bf{k}}}}}_{\mathrm{s}}}({{{\bf{G}}}})\right|}^{2},$$

found from a set (here, 100) of MP2 calculations with different twist angles. One twist angle is chosen from this set and run at the CCSD level.

This approach is based on the idea that the single twist angle then represents the average transition structure factor in a way that can be transferred across different methods. In particular, our previous work on model systems has shown the transferability of systems to CCSD^28^ and full configuration interaction quantum Monte Carlo^30^. MP2 theory is a natural choice because it is used as the starting point to a CCSD calculation but, in principle, other correlated methods could be used. The key feature that determines a good method to use is a method that well represents the variation in the structure factor with changing offset. In replacing an averaged quantity with a single point, we are suggesting that the mean-value theorem for integration will be approximately valid even in the presence of the ∑_**G**_ in equation (7).

### Codes and methods

All calculations were performed using VASP 5.4/VASP 6 (refs. ^51,52^) and the projector augmented-wave method in a plane wave basis set^53^. The corrections for the finite-size effects for lithium and silicon were performed using cc4s interfaced with the VASP code^7^. Twist averaging was used to obtain the HF data. All other calculations were run using the sfTA method. Canonical HF orbitals were used for all MP2 calculations. For coupled cluster, we used approximate natural orbitals, estimated from MP2 natural orbitals^54^, for the lithium basis-set convergence calculations and all of the silicon phase calculations. The rest of the calculations used canonical HF orbitals.

### Calculation details

All calculations require a plane wave energy cutoff (ENCUT), an auxilliary plane wave basis-set cutoff (ENCUTGW), a *k*-point mesh, and a number of bands in the correlated part of the calculation NBANDS. All calculations used a Perdew–Burke–Ernzerhof (PBE) pseudopotential except the 32-atom sodium supercell calculations in Fig. 1, which used a Ceperley–Alder (CA) pseudopotential.

We used an ENCUT of 400 and an ENCUTGW of 150 eV for the 32-atom sodium supercell calculations in Fig. 1. The basis set was truncated to 48 orbitals per *k*-point (using the NBANDS input); this calculations was a 1 × 1 × 1 *k*-point mesh supercell.

The ten systems shown in Fig. 2 were run on a *k*-point mesh of 2 × 2 × 2 with a truncated basis set of 32 orbitals per *k*-point. For the carbon, lithium fluoride, magnesium oxide and silicon carbide calculations an ENCUT of 400 and an ENCUTGW of 300 were used for the energy cutoffs. For the germanium, lithium hydride and cubic-boron nitride calculations an ENCUT of 300 was used. The germanium calculations used an ENCUTGW of 250 and lithium hydride and cubic-boron nitride calculations used an ENCUTGW of 200. The potassium and aluminum calculations used an ENCUT of 200 and an ENCUTGW of 150. The sodium calculations used an ENCUT of 80 and an ENCUTGW of 60. These correspond to recommended cutoffs listed in the potential files provided in VASP.

The lithium and silicon calculations used an ENCUT of 400 eV. For these calculations, ENCUTGW was changed to 400 eV to ensure the initial basis set was large enough that the number of orbitals designated by the NBANDS input was the limiting factor for truncating the basis set in all calculations.

Different *k*-point meshes were used for the TDL extrapolations. For lithium, *k*-point meshes of 2 × 2 × 2, 3 × 3 × 3, and 4 × 4 × 4 were used. Silicon had *k*-point meshes of 2 × 2 × 2 and 3 × 3 × 3. Each phase used an NBANDS of 48 except lithium bcc. The lithium bcc calculations used a different NBANDS of 24 orbitals per *k*-point which is the same number of *k*-points per atom as fcc. To calculate the complete basis-set energy for each lithium and silicon phase, a 2 × 2 × 2 *k*-point mesh was used with NBANDS changed to give 16, 24, 32, 40 and 48 orbitals per *k*-point.

Out of the ~10,000 MP2 calculations performed at random individual twist angles, a small number (three) of lithium fcc MP2 calculations (at 3 × 3 × 3) did not converge at the HF level due to small gap effects. In these cases, a new twist angle was chosen at random and this is unlikely to greatly affect twist angle selection. The convergence thresholds used for calculations are tabulated in the institutional repository referenced in the Data Availability section.

### Lattice information

The following systems had a set volume, *V*, for each unit cell as follows: sodium, *V* = 70.193 Å^3^; potassium, *V* = 151.25 Å^3^; aluminum, *V* = 63.1399 Å^3^; germanium, *V* = 44.95 Å^3^; lithium hydride, *V* = 16.002 Å^3^; cubic-boron nitride, *V* = 11.59 Å^3^; carbon, *V* = 11.2131 Å^3^; lithium fluoride, *V* = 15.67 Å^3^; magnesium oxide, *V* = 18.38 Å^3^; silicon carbide, *V* = 20.52 Å^3^. Each of these systems was run using two atoms per unit cell, except aluminum, which used four atoms per unit cell. For each system list above, the phase has been included in the label in Fig. 2 using the Strukturbericht symbols, with A1 = face-centered-cubic, A2 = body-centered-cubic phase, A4 = diamond, B1 = rock-salt and B3 = zinc-blende. The 32-atom sodium supercell in Fig. 1 was set to a volume of *V* = 1202.424 Å^3^. The equilibrium lattice constants, *a*, for β-Sn silicon and diamond silicon (which are *a* = 4.9 Å and *a* = 5.761 Å, respectively) were scaled by factors in the range 0.85 to 1.1 to produce a range of volumes. Lithium structure information, including equilibrium lattice constants (*a* = 3.436 for bcc and *a* = 4.305 for fcc), were obtained from the NOMAD Encyclopedia database^55^.

### Birch–Murnaghan fits

Energies for Fig. 4 and Table 1 were calculated from TA-HF and sfTA-CCSD-FS, followed by extrapolation to the TDL. The fit in Fig. 4 is a standard Birch–Murnaghan equation of state curve, yielding the parameters in the first eight rows of Table 1. From these, the energy difference between the two minima (Δ*E*) was calculated and the slope of a common tangent between Birch–Murnaghan fits of the two phases was used to find *P*_t_.

We had to apply the following corrections to make the data Table 1 consistent between studies: (1) all Δ*E* (and subsequently *P*_t_) with the exception of HF and diffusion Monte Carlo with an empirical pseudopotential correction (DMC + EMP-pp) contain a core-polarization correction of 30 meV per atom taken from Alfè and colleagues^19^ whether added by us or by previous authors. For DMC + EMP-pp^21^, we use the data including the core-polarization corrections referenced in their paper. (2) The fully corrected transition pressures (*P*_t_ vib.) contain the corrections for zero-point energy, finite-temperature vibrational effects, and core-polarization. We include these vibrational correction terms in CCSD following the numbers from Alfè and co-workers^19^.

Finally, for interested readers, a detailed exploration of the comparisons between these data and state-of-the-art DFT numbers are given by Hennig et al.^18^ and Xiao and colleagues^41^.

### Numerical analysis

Graphs were plotted using matplpotlib with Python 3.7.3. Box-and-whisker plots are constructed using matplotlib.pyplot.boxplot. For the extrapolations to the TDL and the Birch–Murnaghan fits the numpy and scipy libraries were used with Python 3.7.3. Error bars on the TDL extrapolations reflect standard error in the fit parameters.

### Supplementary information

Supplementary Information Supplementary Discussion, and Tables 1 and 2.

### Source data

Source Data Fig. 1 Statistical source data for Fig. 1 consisting of three data excels and a README.

Source Data Fig. 2 Statistical source data for Fig. 2 consisting of two data excels and a README.

Source Data Fig. 3 Statistical source data for Fig. 3 consisting of a data excel and a README.

Source Data Fig. 4 Statistical source data for Fig. 4 consisting of a data excel and a README.

## Data Availability

The Supplementary Information contains information on how the data were assembled into total energies for silicon and lithium in ‘Assembling the total energy’, with examples shown in Supplementary Tables 1 and 2. Data for this study was generated using VASP 5.4/6 and the sfTA code at Zenodo and https://github.com/shepherd-group/sfTA (ref. ^56^). The inputs and datasets from this study are available at the Iowa Research Online (IRO) repository at 10.25820/data.006153 (ref. ^57^). We have also written a guide for how readers can re-use our data in the Supplementary Section entitled ‘Where to find/how to use data’. Source Data are provided with this paper.

## Code availability

The sfTA scripts to calculate the twist angles, including an example, appear at the Zenodo repository^56^ (10.5281/zenodo.5553436) and on GitHub (https://github.com/shepherd-group/sfTA). We have also included a utility script for using VASP. This repository will be maintained after publication and prospective future users are invited to contact the corresponding author with any additional requests. This is an open-source code released under an MIT licence. The modifications of the VASP code used to calculate the transition structure factor and CCSD energies were made in a local repository based on VASP 5.4/VASP 6. The code CCSD.F will be merged into a later release of VASP. VASP and its source code is software available for purchase. The pseudopotentials used for these calculations are distributed with VASP.
