# Supplementary material for: A shortcut to the thermodynamic limit for quantum many-body calculations of metals
Source: Nat Comput Sci. 2021 Dec 16;1(12):801–8. doi: 10.1038/s43588-021-00165-1 (PMC10766528; doi:10.1038/s43588-021-00165-1)
Supplement: Supplementary file 1 — Supplementary Discussion, and Tables 1 and 2. [file 43588_2021_165_MOESM1_ESM.pdf]

nature portfolio

https://doi.org/10.1038/s43588-021-00165-1

Supplementary information

A shortcut to the thermodynamic limit for quantum many-body calculations of metals

In the format provided by the authors and unedited

Supplementary Information for "A shortcut to the thermodynamic limit for quantum many-body calculations of metals"

Tina N. Mihm,[1] Tobias Schäfer,[2] Sai Kumar Ramadugu,[1]
Laura Weiler,[1] Andreas Grüneis,[2] and James J. Shepherd[1,*]

[1] *Department of Chemistry, University of Iowa, Iowa City, Iowa, 52242, USA*

[2] *Institute for Theoretical Physics, TU Wien, Wiedner Hauptstraße 8-10/136, 1040 Vienna, Austria*

(Dated: October 15, 2021)

* james-shepherd@uiowa.edu

WHERE TO FIND/HOW TO USE DATA

The following section provides details on what can be done with the data and information provided in this paper. This is organized by activities that we anticipated readers would want to do. Readers may wish to:

- Use CCSD benchmark data for comparison (Si or Li): Excel files in the Source Data section contain the raw data from Figs. 3, 4, and Table I. To plot smooth curves, use the standard Birch-Murnighan equation of state with the parameters provided in Table I.

- Repeat the energy assembly from raw data (Si or Li): The overall protocol is given in the Results section of the main manuscript. Two examples are shown in the Supporting Information in the section: "Assembling the total energy". Additional raw data for further analysis can then be found in the data repository linked in the main text under "Data availability".

- Repeat the validity check on 10 systems from Fig 2: Processed energy data for plotting only can be found in the Excel files in the Source Data. Raw energy data can be found in the repository linked under "Data availability" which can be used to check the subtraction of the Γ-point ($\mathbf{k}_s = \mathbf{0}$) energy.

- Run VASP calculations to... (note that this will require a future public release of VASP incorporating our algorithms, see "Code availability" statement in the main manuscript).

  - Repeat calculations in this paper: Input options are provided per system in the repository linked under "Data Availability". A high level summary of the input options appears in both Results and Methods sections of the main manuscript.

  - Calculate sfTA-CCSD for any system: Follow the protocol in the main manuscript under "Methods". Our scripts are provided in the sfTA Github repository linked in the "Code availability" statement to set up/run a VASP sfTA-CCSD calculation and perform the post-processing required to find the special twist angle.

  - Calculate energy assemblies such as those outlined in the main manuscript: The end-to-end protocol is provided in the main text in the Results section. Guidance on what parameters to be chosen appears in the Results and Methods sections.

- Replicate sfTA in another code: The protocol for this is outlined in the main manuscript. This requires code modifications which allow for twist averaging and the calculation of the transition structure factor. Example transition structure factors are given in an Excel table in the sfTA Github repository linked in the in the "Code availability" statement.

- Replicate energy assembly with another code: The end-to-end protocol is provided in the main text in the Results section.

ASSEMBLING THE TOTAL ENERGY

Supplementary Table I shows the workflow used to obtain the energies shown in the paper for the sfTA-CCSD calculations for body-centered cubic (bcc) and face-centered cubic (fcc) phase of lithium.

The first five steps, step A – E, show the twist averaged Hartree–Fock calculations for a range of k-point grids ranging from $2 \times 2 \times 2$ to $6 \times 6 \times 6$. Each of calculations were run over 100 randomly selected twist angles and averaged to get the final twist averaged Hartree–Fock (TA-HF) energies. These energies were then extrapolated over (using a linear $1/N_k$ extrapolation) to get the TA-HF energy at the thermodynamic limit (TDL) shown in step F; in particular, C, D, and E were included in the extrapolation range because these lay on a straight line.

Next, we ran sfTA-CCSD calculations following the protocol outlined in the paper for of lithium at three different k-point grid sizes, $2 \times 2 \times 2$, $3 \times 3 \times 3$, and $4 \times 4 \times 4$. The results are shown in steps G – I. We note that three Li fcc MP2 calculations (at $3 \times 3 \times 3$) did not converge at the HF level due to small gap effects and a new random twist angle was selected. After the sfTA-CCSD energies were obtained, finite size corrections were calculated using the method outlined in Ref. 1. The resulting finite sized corrected sfTA-CCSD energies (sfTA-CCSD-FS) can be seen in steps J – L in the table.

We then ran sfTA-CCSD calculations on a range of basis sets from 16 to 48 orbitals per

k-point. All of these basis sets were run on a $2 \times 2 \times 2$ k-point grid for both phases of lithium and the resulting energies can be seen in steps M – Q in the table. The $2 \times 2 \times 2$ size is chosen to be the largest system that can be afforded given the basis set range. Up to 48 orbitals per k-point is a reasonably common basis set size. We then obtained the complete basis set (CBS) limit shown in step R through extrapolating the last three energies (steps O – Q) using a $1/M$ power-law (where M is the number of bands) and used the resulting CBS limit to calculate a basis set correction. This correction, shown in step S, was obtained through taking the difference between the CBS limit in step R and the sfTA-CCSD energy for the $2 \times 2 \times 2$ k-point grid in step J. This provided a basis set correction for the sfTA-CCSD-FS energies, which was then applied uniformly across the k-point grids as shown in steps T – V.

Finally, the sfTA-CCSD-FS basis set corrected energies were extrapolated to the TDL (step W). This was then added to the TA-HF energy in step F to get the total energy at the thermodynamic limit for the two phases of lithium.

The same workflow is shown for the two silicon phases, β-Sn and diamond, in Supplementary Table II for the equilibrium volumes. There are two main differences between the two workflows. First, the Hartree–Fock (HF) calculations only goes up to a $5 \times 5 \times 5$ k-point grid for the silicon phases. Second, the finite-sized corrected data is extrapolated before the basis set correction is added. The CPP correction has also been included in the β-tin Si table to ensure the total energy reflect what is shown in Fig 4 in the paper.

[1] T. Gruber, K. Liao, T. Tsatsoulis, F. Hummel, and A. Grüneis, Physical Review X **8**, 021043 (2018).

Supplementary Table I. An example calculation showing the steps taken to get the final total energies in the paper for the two phases of lithium.

Step	Value (eV/atom)	Description	Derived from?
A	-2.8003276	Li-bcc Twist-averaged 222 HF energy	
B	-2.451186	Li-bcc Twist-averaged 333 HF energy	
C	-2.3449499	Li-bcc Twist-averaged 444 HF energy	
D	-2.2942773	Li-bcc Twist-averaged 555 HF energy	
E	-2.2658333	Li-bcc Twist-averaged 666 HF energy	
F	-2.234801	Li-bcc Extrapolated TA HF energy	Extrapolate C thru E
G	-0.6161585	Li-bcc sfTA 222 CCSD energy	
H	-0.729276	Li-bcc sfTA 333 CCSD energy	
I	-0.7721534	Li-bcc sfTA 444 CCSD energy	
J	-0.71005	Li-bcc sfTA 222 CCSD-FS energy	
K	-0.796835	Li-bcc sfTA 333 CCSD-FS energy	
L	-0.81828	Li-bcc sfTA 444 CCSD-FS energy	
M	-0.5958742	Li-bcc sfTA 222, nbands 16 CCSD energy	
N	-0.6087229	Li-bcc sfTA 222, nbands 24 CCSD energy	
O	-0.6132431	Li-bcc sfTA 222, nbands 32 CCSD energy	
P	-0.6156514	Li-bcc sfTA 222, nbands 40 CCSD energy	
Q	-0.6170119	Li-bcc sfTA 222, nbands 48 CCSD energy	
R	-0.6246464	Li-bcc Extrapolated 222 sfTA-CCSD CBS limit energy	Extrapolate O thru Q
S	0.08540359	Li-bcc sfTA-CCSD basis set correction	Difference between R and J
T	-0.5307549	Li-bcc basis set corrected 222 sfTA-CCSD-FS energy	Add S and J
U	-0.6438724	Li-bcc basis set corrected 333 sfTA-CCSD-FS energy	Add S and K
V	-0.6867498	Li-bcc basis set corrected 444 sfTA-CCSD-FS energy	Add S and L
W	-0.7482019	Li-bcc basis set corrected Extrapolation sfTA-CCSD-FS energy	Extrapolate T thru V
Total energy	-2.9830028		F + W

Step	Value (eV/atom)	Description	Derived from?
A	-2.5454282	Li-fcc Twist-averaged 222 HF energy	
B	-2.3707127	Li-fcc Twist-averaged 333 HF energy	
C	-2.2942956	Li-fcc Twist-averaged 444 HF energy	
D	-2.2590706	Li-fcc Twist-averaged 555 HF energy	
E	-2.2416026	Li-fcc Twist-averaged 666 HF energy	
F	-2.2201318	Li-fcc Extrapolated TA HF energy	Extrapolate C thru E
G	-0.6778486	Li-fcc sfTA 222 CCSD energy	
H	-0.7562848	Li-fcc sfTA 333 CCSD energy	
I	-0.78669	Li-fcc sfTA 444 CCSD energy	
J	-0.7586925	Li-fcc sfTA 222 CCSD-FS energy	
K	-0.8058825	Li-fcc sfTA 333 CCSD-FS energy	
L	-0.81884	Li-fcc sfTA 444 CCSD-FS energy	
M	-0.6159589	Li-fcc sfTA 222, nbands 16 CCSD energy	
N	-0.6588028	Li-fcc sfTA 222, nbands 24 CCSD energy	
O	-0.675143	Li-fcc sfTA 222, nbands 32 CCSD energy	
P	-0.6834531	Li-fcc sfTA 222, nbands 40 CCSD energy	
Q	-0.6883925	Li-fcc sfTA 222, nbands 48 CCSD energy	
R	-0.7151286	Li-fcc Extrapolated 222 sfTA-CCSD CBS limit energy	Extrapolate O thru Q
S	0.04356392	Li-fcc sfTA-CCSD basis set correction	Difference between R and J
T	-0.7151286	Li-fcc basis set corrected 222 sfTA-CCSD-FS energy	Add S and J
U	-0.7623186	Li-fcc basis set corrected 333 sfTA-CCSD-FS energy	Add S and K
V	-0.7752761	Li-fcc basis set corrected 444 sfTA-CCSD-FS energy	Add S and L
W	-0.7832432	Li-fcc basis set corrected Extrapolation sfTA-CCSD-FS energy	Extrapolate T thru V
Total energy	-3.003375		F + W

Supplementary Table II. An example calculation showing the steps taken to get the final total energies in the paper for volume points close to equilibrium for the two phases of silicon.

Letter	Value	Description	Derived from?
A	-9.346775785	Si-btin Vol 15.22 Twist-averaged 222 HF energy	
B	-8.541213565	Si-btin Vol 15.22 Twist-averaged 333 HF energy	
C	-8.34459737	Si-btin Vol 15.22 Twist-averaged 444 HF energy	
D	-8.233555035	Si-btin Vol 15.22 Twist-averaged 555 HF energy	
E	-8.165078618	Si-btin Vol 15.22 Extrapolated TA HF energy	Extrapolate B thru D
F	-2.504236909	Si-btin Vol 15.22 Basis set 222, nband 16 sfTA-CCSD energy	
G	-3.098034256	Si-btin Vol 15.22 Basis set 222, nband 24 sfTA-CCSD energy	
H	-3.336735545	Si-btin Vol 15.22 Basis set 222, nband 32 sfTA-CCSD energy	
I	-3.466829245	Si-btin Vol 15.22 Basis set 222, nband 40 sfTA-CCSD energy	
J	-3.546594575	Si-btin Vol 15.22 Basis set 222, nband 48 sfTA-CCSD energy	
K	-3.969061504	Si-btin Vol 15.22 Basis set 222, CBS limit sfTA-CCSD energy	Extrapolate H thru J
L	-0.422466929	Si-btin Vol 15.22 Basis set 222, basis set correction	Difference between K and J
M	-3.573484106	Si-btin vol 15.22 222 sfTA-CCSD energy	
N	-3.891664793	Si-btin vol 15.22 333 sfTA-CCSD energy	
O	-3.893529106	Si-btin vol 15.22 222 sfTA-CCSD-FS energy	
P	-4.085645	Si-btin vol 15.22 333 sfTA-CCSD-FS energy	
Q	-4.166535903	Si-btin vol 15.22 extrapolated sfTA-CCSD-FS energy	Extrapolate O thru P
R	-0.03	CPP correction	
Total energy	-12.78408145		Q + L + R + E

Letter	Value	Description	Derived from?
A	-10.17737372	Si-diamond Vol 20.31 Twist-averaged 222 HF energy	
B	-9.676165065	Si-diamond Vol 20.31 Twist-averaged 333 HF energy	
C	-9.552566875	Si-diamond Vol 20.31 Twist-averaged 444 HF energy	
D	-9.517043115	Si-diamond Vol 20.31 Twist-averaged 555 HF energy	
E	-9.469832759	Si-diamond Vol 20.31 Extrapolated TA HF energy	Extrapolate B thru D
F	-2.172735006	Si-diamond Vol 20.31 Basis set 222, nband 16 sfTA CCSD energy	
G	-2.711179923	Si-diamond Vol 20.31 Basis set 222, nband 24 sfTA CCSD energy	
H	-2.94576508	Si-diamond Vol 20.31 Basis set 222, nband 32 sfTA CCSD energy	
I	-3.071299634	Si-diamond Vol 20.31 Basis set 222, nband 40 sfTA CCSD energy	
J	-3.144164645	Si-diamond Vol 20.31 Basis set 222, nband 48 sfTA CCSD energy	
K	-3.545236679	Si-diamond Vol 20.31 Basis set 222, CBS limit sfTA CCSD energy	Extrapolate H thru J
L	-0.401072034	Si-diamond Vol 20.31 Basis set 222, basis set correction	Difference between K and J
M	-3.150708803	Si-diamond vol 20.31 222 sfTA-CCSD energy	
N	-3.322491308	Si-diamond vol 20.31 333 sfTA-CCSD energy	
O	-3.414538803	Si-diamond vol 20.31 222 sfTA-CCSD-FS energy	
P	-3.446345	Si-diamond vol 20.31 333 sfTA-CCSD-FS energy	
Q	-3.459737083	Si-diamond vol 20.31 extrapolated sfTA-CCSD-FS energy	Extrapolate O thru P
Total energy	-13.33064188		Q + L + E
